# *Tintelnotia destructans* Invasive Rhinosinusitis in an Immunocompromised Child: A Case Report and Review of the Literature

**DOI:** 10.1093/ofid/ofag526

**Published:** 2026-08-21

**Authors:** Manon Jaboyedoff, Frederic Lamoth, Claire Bertelli, Jizhou Li, Estelle Dubruc, Frida Rizzati, Antoine Reinhard, Raffaele Renella, Pierre Alex Crisinel

**Affiliations:** Unit of Pediatric Infectious Diseases and Vaccinology, Service of Pediatrics, Department Women-Mother-Child, Lausanne University Hospital and University of Lausanne, Lausanne, Switzerland; Institute of Microbiology, Lausanne University Hospital and University of Lausanne, Lausanne, Switzerland; Infectious Diseases Service, Department of Medicine, Lausanne University Hospital and University of Lausanne, Lausanne, Switzerland; Institute of Microbiology, Lausanne University Hospital and University of Lausanne, Lausanne, Switzerland; Institute of Microbiology, Lausanne University Hospital and University of Lausanne, Lausanne, Switzerland; Infectious Diseases Service, Department of Medicine, Lausanne University Hospital and University of Lausanne, Lausanne, Switzerland; Institute of Pathology, Lausanne University Hospital and University of Lausanne, Lausanne, Switzerland; Pediatric Intensive Care Unit, Service of Pediatrics, Department Women-Mother-Child, Lausanne University Hospital and University of Lausanne, Lausanne, Switzerland; Department of Otorhinolaryngology–Head and Neck Surgery, Lausanne University Hospital and University of Lausanne, Switzerland; Pediatric Hematology-Oncology Unit, Service of Pediatrics, Department Women-Mother-Child, Lausanne University Hospital and University of Lausanne, Lausanne, Switzerland; Unit of Pediatric Infectious Diseases and Vaccinology, Service of Pediatrics, Department Women-Mother-Child, Lausanne University Hospital and University of Lausanne, Lausanne, Switzerland

**Keywords:** aplastic anemia, fungal sinusitis, immunocompromised host, invasive fungal infection, opportunistic fungal infection

## Abstract

*Tintelnotia destructans* is a recently described fungus within the family Phaeosphaeriaceae, previously reported only in superficial human infections such as keratitis, onychomycosis, and noninvasive sinus fungus ball. We describe the first case of invasive rhinosinusal infection due to this pathogen in a child with severe aplastic anemia. Diagnosis was established by histopathology showing submucosal fungal invasion and broad-range fungal polymerase chain reaction targeting 18S rRNA identifying *T. destructans*. Combined endoscopic surgical debridement, antifungal therapy (liposomal amphotericin B and isavuconazole), and immune reconstitution after emergency haploidentical hematopoietic stem cell transplant ultimately led to complete recovery without recurrence 18 months after treatment, despite continued therapeutic immunosuppression for graft-vs-host disease. This case expands the known pathogenic spectrum of *T. destructans*, demonstrating its potential to cause invasive disease in severely immunocompromised hosts. Advances in molecular diagnostic tools may expand the spectrum of fungi causing invasive fungal infections by identification of novel pathogenic species.

A 10-year-old female with a 9-month history of severe aplastic anemia refractory to immunosuppressive therapy (cyclosporin, steroids, and antithymocyte globulin; EWOG-MDS/SAA 2010 protocol) and awaiting hematopoietic stem cell transplantation (HSCT) was diagnosed with proven pulmonary mucormycosis due to *Rhizomucor* sp. The diagnosis was supported by a thoracic computed tomography scan showing left lower-lobe consolidation with central necrosis, a positive *Rhizomucor* sp polymerase chain reaction (PCR) result in bronchoalveolar lavage (BAL), and histopathologic examination of a pulmonary resection specimen demonstrating numerous mycelial filaments morphologically compatible with Mucorales. Liposomal amphotericin B (5 mg/kg) was initiated at the time when mucormycosis was first suspected (Figure 1). She was receiving anti-infective prophylaxis with trimethoprim-sulfamethoxazole, valaciclovir, levofloxacin, and posaconazole, with regular therapeutic drug monitoring. She lived on a farm in a rural area of Switzerland. In the weeks preceding pulmonary mucormycosis, she had presented fever, pansinusitis, and tonsillar hypertrophy attributed to Epstein-Barr virus reactivation after an extensive workup.

**Figure 1.**
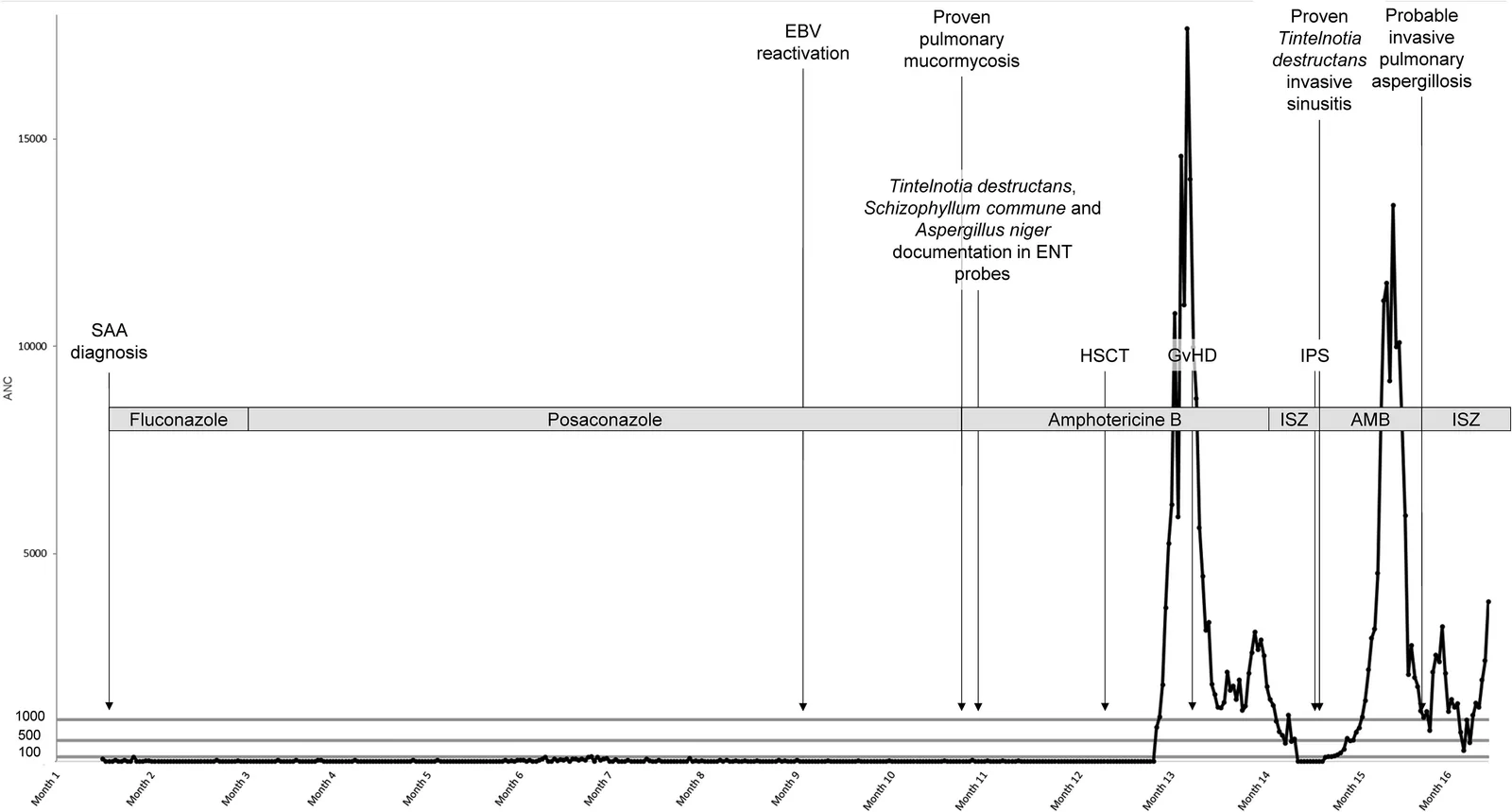
Clinical course. The black line represents absolute neutrophil count over time. The gray bar shows antifungal treatments over time. AMB, amphotericin B; EBV, Epstein-Barr virus; ENT, ear, nose, and throat; GvHD, graft-vs-host disease; HSCT, hematopoietic stem cell transplantation; IPS, idiopathic pulmonary syndrome; ISZ, isavuconazole; SAA, severe aplastic anemia.

Sinus exploration was conducted in the context of radiologic evidence of sinusitis and concomitantly with pulmonary lobectomy of the involved lobe performed 4 days after mucormycosis was suspected, and it showed 2 lesions: an extramucosal lesion in the right sphenoid sinus, suspected to be a fungus ball, and a superficial white mucosal lesion in the posterior nasal cavity. Fungal cultures of the presumed fungal ball yielded 1 colony of *Schizophyllum commune* and 1 of *Aspergillus niger*, as identified by matrix-assisted laser desorption/ionization–time-of-flight mass spectrometry. Histopathologic examination of the superficial white lesion showed respiratory-type mucosa with mild chronic inflammation and an area of necrosis partially calcified, containing broad short-branched hyphae positive for Grocott-Gomori methenamine silver and periodic acid–Schiff stains (Figure 2). Fungal culture of this lesion on Sabouraud dextrose (2%) agar grew 21 to 25 colonies of mold showing typical flask-shaped pycnidia, which was identified as *Tintelnotia destructans* by broad fungal PCR targeting the 26S rRNA gene, as no acceptable identification was obtained by matrix-assisted laser desorption/ionization–time-of-flight mass spectrometry [1] (Figure 2). No *Rhizomucor* sp was identified in the sinusal samples. At that stage, the 3 fungal pathogens were assessed as representing superficial sinus infection, and amphotericin B therapy initiated for proven pulmonary mucormycosis was continued. Unrelated allogeneic HSCT was performed 2 months after the pulmonary *Rhizomucor* sp diagnosis, following extensive multidisciplinary discussions and under continued antifungal therapy. The posttransplant course was complicated by severe acute digestive and cutaneous graft-vs-host disease (grade 4) and by idiopathic pulmonary syndrome. These conditions were treated by immunosuppression by multiple agents (steroids, cyclosporine, tacrolimus, tocilizumab, ruxolitinib). Six weeks after HSCT and 3 months after start of amphotericin B, antifungal therapy was switched to isavuconazole (200 mg once daily after initial loading doses, with regular therapeutic drug monitoring) due to amphotericin B–induced nephrotoxicity.

**Figure 2.**
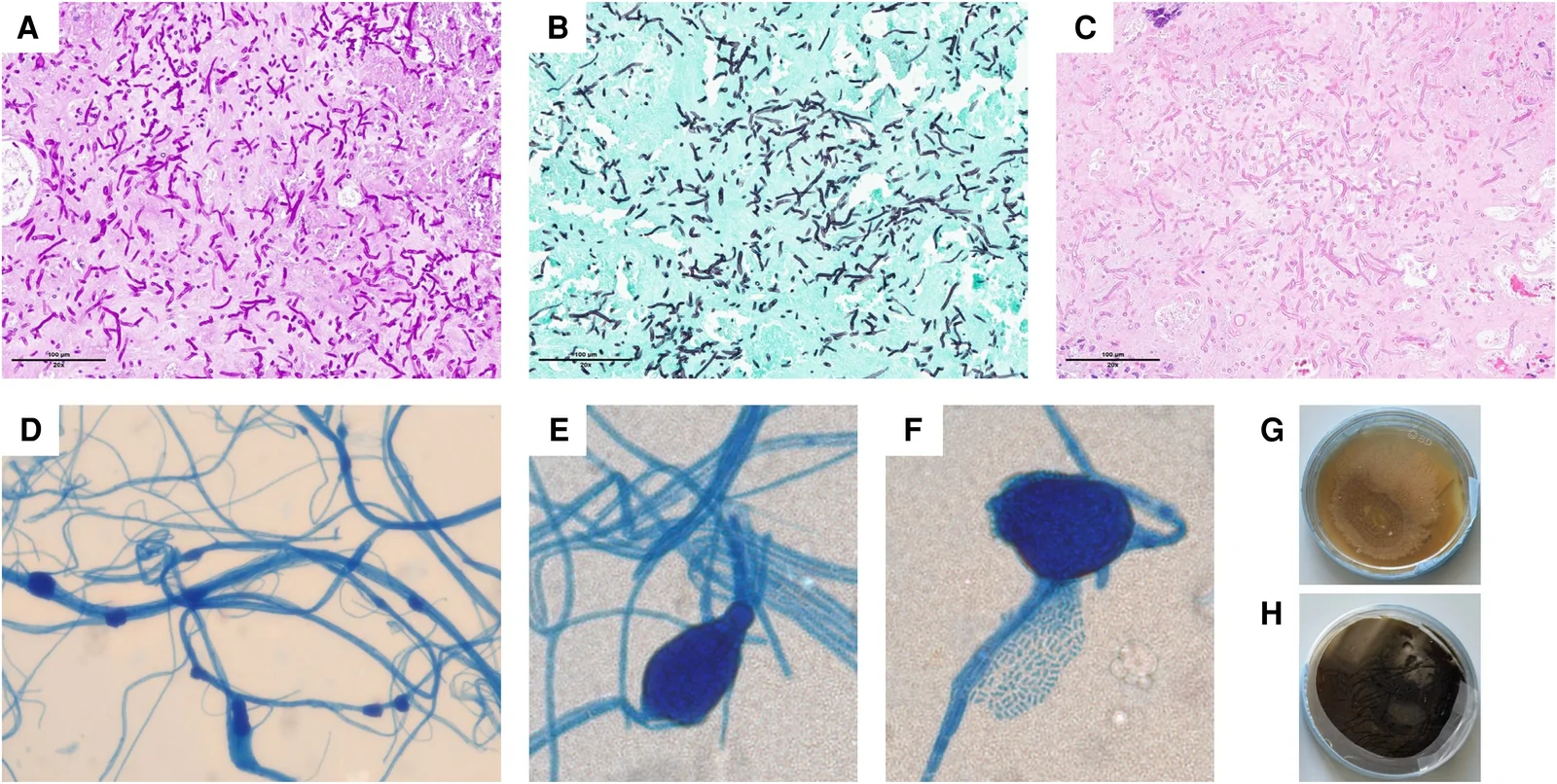
Histopathologic findings (sinusal mucosa) and microscopy of *Tintelnotia destructans* after lactophenol cotton blue stain. *A*, Periodic acid–Schiff stain shows fungal hyphae (20×). *B*, Grocott-Gomori methenamine silver stain shows fungal hyphae (20×). *C*, Hematoxylin and eosin stain reveals an area of necrosis with short branched hyphae (20×). *D*, Filaments and fruiting bodies at 100× magnification. *E* and *F*, Flask-shaped pycnidia and released conidia at 1000× magnification. *G* and *H*, Growth of *T. destructans* on Sabouraud agar and potato agar plates at 30 °C.

Two months after HSCT and 2 weeks after switching to isavuconazole, the patient developed nasal pain. Magnetic resonance imaging showed pansinusitis and a nodule of the nasal septum. Computed tomography scan revealed lytic lesions of the nasal bone, suggesting progression of fungal rhinosinusitis (Figure 3). Upper airways exploration with local endoscopic debridement was performed. Direct microscopy of bone biopsy revealed mycelial elements. Fungal culture remained negative, but broad fungal PCR targeting 18S rRNA detected 1400 copies/mL of *Tintelnotia* sp [1]. Histopathology confirmed invasive fungal sinusitis with extension to the bone tissue. Due to limited evidence on the efficacy of isavuconazole against *T. destructans* and considering previously reported low minimal inhibitory concentrations (MICs) for amphotericin B against *T. destructans*, antifungal therapy was switched back to amphotericin B (Table 1). One month later, isavuconazole was resumed following a new diagnosis of probable invasive pulmonary aspergillosis (based on host factors, positive galactomannan in BAL, *Aspergillus fumigatus* growth in BAL culture, compatible pulmonary imaging findings, and negative *Rhizomucor* BAL PCR and culture results) and concerns about recurrent amphotericin B toxicity. Antifungal therapy with isavuconazole was continued until immune reconstitution for a total antifungal treatment duration of 16 months.

**Figure 3.**
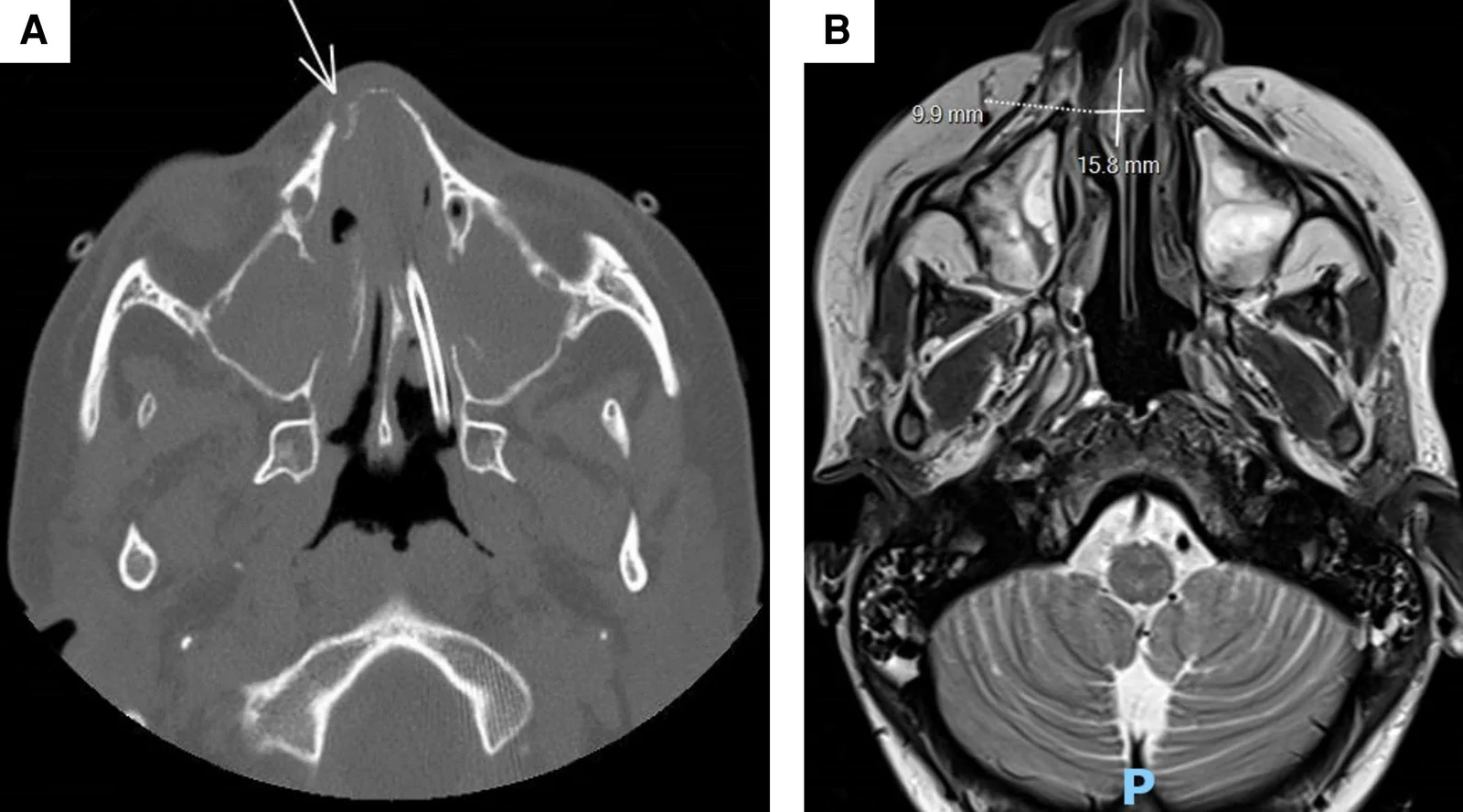
Radiologic findings. *A*, Computed tomography scan of the paranasal sinuses shows osteolytic lesions of the nasal bones. *B*, Magnetic resonance imaging illustrates a nodule of the nasal septum.

**Table 1.** Summary of Published Antifungal Minimum Inhibitory Concentrations (mg/L) of *Tintelnotia destructans*

First Author	No. of Strains Tested	ANI	AMB	CAS	FCZ	ISZ	ITZ	PCZ	VCZ	TBF	MGX
Present case	1	…	1–2	…	…	16	…	1	1–2	…	>2
Sevestre [2]	1	…	0.25	…	…	>32	…	0.25	0.5	…	…
Romero-Barranca [3]	1	0.015	0.25	0.25	…	8	0.5	0.25	1	0.25	…
Kaufmann [4]	1	>32	0.094	>32	256	4	2	0.380	0.125	S	…
Roels [5]	1	…	0.125	…	…	…	0.03	…	0.5	…	…
Tan [6]	1	>8	1	>8	16	…	0.12	0.25	0.5	…	…
Behrens-Baumann [7]	1	…	…	…	16	…	1	1	1	0.12	…
Habbe [8]	1	0.5	0.5	<0.016	…	4	0.25	0.125	…	…	…
Ahmed [9]	4	…	1	16^a^	16	…	1	1	1	0.125	…
Walther [10]	3	…	0.5–1	≤0.016–0.25	…	4–8	0.25–0.5	0.06–0.125	0.5–1	…	…

Abbreviations: AMB, amphotericin B; ANI, anidulafungin; CAS, caspofungin; FCZ, fluconazole; ISZ, isavuconazole; ITZ, itraconazole; MGX, manogepix; PCZ, posaconazole; S, susceptible; TBF, terbinafine; VCZ, voriconazole.

^a^Minimum effective concentration.

Three years after HSCT and 18 months after cessation of antifungal therapy for *T. destructans* rhinosinusitis, the patient shows no signs of recurrence and has no facial deformity.

Antifungal susceptibility testing of the *T. destructans* isolate was retrospectively performed in duplicates according to the protocol of the European Committee on Antimicrobial Susceptibility Testing. MICs were as follow: amphotericin B, 1 to 2 mg/L; voriconazole, 1 to 2 mg/L; posaconazole, 1 mg/L; isavuconazole, 16 mg/L; manogepix, >2 mg/L.

## DISCUSSION

We report here the first case description of *T. destructans* invasive rhinosinusitis.

Invasive fungal rhinosinusitis is a severe infection of the nasal cavity and paranasal sinuses, typically occurring in individuals with impaired host defenses. In adults, uncontrolled diabetes mellitus is the most common predisposing condition, followed by hematologic malignancies [11]. In children, hematologic malignancies and aplastic anemia predominate [12]. Mortality approaches 50% in both age groups, underscoring the need for early diagnosis [11, 12].

Fungi of the order Mucorales (*Lichtheimia* sp, *Rhizomucor* sp, *Mucor* sp, *Rhizopus* sp) and *Aspergillus* spp are the most frequent pathogens responsible for invasive fungal rhinosinusitis. *Fusarium* and *Alternaria* species are less frequently involved [12]. Treatment comprises antifungal therapy and surgical debridement to remove necrotic tissue and decrease fungal load. In the present case, although multiple fungal species were isolated during the clinical course, only *T. destructans* was associated with histopathologic evidence of tissue invasion in the sinonasal compartment.

No case of *Tintelnotia* spp invasive infection has been described in the literature. *Tintelnotia* is a genus described in 2017 in the family Phaeosphaeriaceae (order Pleosporales, division Ascomycetes) [13], which mainly consists of phytopathogens. The genus *Tintelnotia* (named after Dr Kathrin Tintelnot, former head of the mycology division of the Robert Koch Institute in Berlin, Germany) contains 2 species: *T. destructans* and *T. opuntiae* [9]. *T. opuntiae* (formerly *Phoma opuntiae*) causes necrotic spots on cactus. The natural niche of *T. destructans* remains unknown, but it has been isolated in several European countries [9, 13].

*T. destructans* recently emerged as a human opportunistic pathogen causing superficial infections, with 8 cases of keratitis, 1 case of noninvasive sinusal fungus ball, 1 case of truncal acne, and 1 case of onychomycosis described in the literature (Table 2) [3–9, 14]. Most patients were immunocompetent, except 3 of them (treated with methotrexate and interleukin 6 receptor antagonist, azathioprine and tumor necrosis factor α inhibitor, and Janus kinase inhibitor, respectively). All cases of keratitis were cured, 3 with surgical debridement and systemic and local antifungal therapy and 5 with systemic (terbinafine and/or voriconazole) and local antifungal therapy only. The patient with a sinusal fungus ball was cured with surgical excision only. The patient with onychomycosis was treated unsuccessfully with local treatments during several months [9].

**Table 2.** Summary of Published Case Reports of *Tintelnotia destructans* Infections

First Author	Year	Age, y/Sex	Type of Infection	Comorbidities	Immunosuppressive Treatment	Antifungal Treatment	Evolution
Present case	2023	9/female	Invasive rhinosinusitis	Severe aplastic anemia	Cyclosporin, steroids, and antithymocyte globulin	Systemic amphotericin B, isavuconazole	No sign of active fungal disease after removal of fungus tissue and 16 mo of treatment
Gano [15]	2025	40/male	Keratitis	Behçet disease	Adalimumab and azathioprine	Systemic terbinafine and voriconazole	Significant improvement after a few weeks
Sevestre [2]	2025	29/female	Sinusal fungus ball	None	None	None	No sign of active fungal disease 2 mo after surgical excision
Romero-Barranca [3]	2023	45/female	Keratitis	None	None	Topical and systemic voriconazole, topical micafungin, topical natamycin, systemic terbinafine	No signs of active fungal disease 1 y later (treatment duration not specified)
Doh [14]	2023	65/male	Truncal acne	Vitiligo	Baricitinib during the preceding 18 wk	Topical ketoconazole	No signs of active fungal disease (treatment duration not specified)
Kaufmann [4]	2021	70/male	Keratitis	Rheumatoid arthritis	Methotrexate, tocilizumab	Ocular natamycin and systemic voriconazole	No sign of active fungal disease after removal of fungus tissue and 1 mo of treatment
Roels [5]	2020	53/female	Keratitis	Herpetic keratitis 6 wk earlier	Ocular dexamethasone during the preceding 6 wk	Ocular voriconazole, oral itraconazole, intracameral injection of amphotericin B	No sign of active fungal disease after penetrating keratoplasty and 6 wk of treatment
Tan [6]	2020	60/female	Keratitis	None. Contact lens wearer	None	Ocular natamycin and voriconazole, intrastromal injection of voriconazole, systemic voriconazole	No sign of active fungal disease after 6 mo of treatment
Behrens-Baumann [7]	2019	Unknown	Keratitis	None. Contact lens wearer, treated for conjunctivitis 1 wk earlier	Ocular prednisolone during the preceding week	Ocular voriconazole and terbinafine, intracameral injections of amphotericin B, systemic voriconazole and terbinafine	No sign of active fungal disease after 4 mo of treatment
Habbe [8]	2017	53/female	Keratitis	None	None	Ocular voriconazole and amphotericin B, intracameral injection of voriconazole, systemic voriconazole	No sign of active fungal disease after penetrating keratoplasty and 2 mo of treatment
Ahmed [9]	2016	56/female	Keratitis	None. Contact lens wearer	None	Ocular voriconazole and terbinafine, intracameral injections of amphotericin B and voriconazole, systemic voriconazole and terbinafine	No sign of active fungal disease after 4 mo of treatment
		45/female	Onychomycosis	None	None	Ungual tioconazole, amorolfine, ciclopirox olamine and salicylic acid/benzoic acid	No improvement

All previously reported cases involved superficial infections.

Our patient presented the typical signs and symptoms of fungal rhinosinusitis. Fungal colonization of her airways likely occurred by inhalation of environmental spores and progressed to infection because of her highly immunosuppressed state. It is possible that the alteration of sinonasal drainage secondary to recent adenotonsillar hypertrophy facilitated progression of the infection. Long exposure to antifungals may have contributed to the selection of *T. destructans*. While the fungus could be grown in culture, identification was achieved only by broad fungal PCR targeting 18S rRNA, which raises the hypothesis that this fungal pathogen may have been underrecognized in the past [1]. The patient initially improved with amphotericin B but experienced a clinical and microbiological relapse after switching to isavuconazole. Data on antifungal susceptibility of *T. destructans* are scarce, and interpretive breakpoints are not available for this fungal species. Antifungal susceptibility testing of the strain showed relatively low MIC for amphotericin B, voriconazole, and posaconazole, while much higher values were observed for isavuconazole, which is in line with previous publications (summarized in Table 1) [2–10]. We also tested the activity of the novel antifungal drug manogepix and found a surprisingly high MIC, which is beyond the concentration range of most pathogenic molds and suggests intrinsic resistance of this species [16]. The fact that the isolate showed high MICs for isavuconazole and relatively low MICs for amphotericin B likely explains the clinical progression from superficial infection to invasive fungal sinusitis after the switch to isavuconazole. The patient was treated with prolonged antifungal therapy (amphotericin B followed by isavuconazole) and local surgical debridement, without signs of active fungal disease more than a year after antifungal treatment interruption. This case illustrates that this pathogen may cause an invasive infection in immunosuppressed hosts. Rapid surgical debridement, antifungal therapy, and immune restoration contributed to the favorable outcome.

## CONCLUSION

We report the first case of an invasive *T. destructans* infection in a child who was immunocompromised. Advances in molecular diagnostic tools may expand the spectrum of fungi causing invasive fungal infections by identification of novel pathogenic species.
